# Becoming a European prisoner: penal reforms and European belonging in Georgia and Estonia

**DOI:** 10.1080/14782804.2024.2324291

**Published:** 2024-03-20

**Authors:** Olga Zeveleva, Costanza Curro

**Affiliations:** a Aleksanteri Institute, University of Helsinki, Helsinki, Finland; b Department of History & Art History, Centre for Conflict Studies, Utrecht University, Utrecht, Netherlands

**Keywords:** Prison reform, criminal justice, Estonia, Georgia, ethnicity

## Abstract

Over the past three decades many former Soviet states introduced reforms to the penal systems they inherited from socialist regimes. In most countries of the post-Soviet space, these reforms have been depicted by policy-makers as projects of ‘Europeanization’. This article investigates the discursive construction of penal reform in Georgia and Estonia after these countries gained independence. We examine images and practices of punishment and answer two questions: how are post-Soviet penal reforms framed by policy-makers and experienced by prisoners? How is Europeanization of punishment understood in the post-Soviet space? We conduct an analysis of policy discourse and of narratives of people who witnessed these changes while serving prison sentences. We treat images of ‘Europe’ as a discursively produced ideational structure, and analyze how different understandings of punishment are linked to, or pitted against, ideas of Europeanness. We locate our study within the framework of critical approaches to European integration, and introduce the study of punishment to these debates. We show that punishment is not simply another case study of the relations between a European ‘centre’ and its ‘peripheries’, but argue rather that images and practices of ‘European punishment’ create new social hierarchies and fractures in post-Soviet societies.

## Introduction

In the past three decades, many former Soviet states have introduced reforms to the penal systems they inherited from socialist regimes. In many countries of the post-Soviet space, penal reforms have been depicted by policy-makers, and often internalized by the public, as projects of prison ‘Europeanization’. This article investigates the discursive construction of penal reform in Georgia and Estonia after these countries gained their independence. We address two questions: how are post-Soviet penal reforms framed by policy-makers and experienced by prisoners? And how is the Europeanization of punishment understood in the post-Soviet space? We answer these questions by examining images and practices of punishment in Georgia and Estonia. We conduct an analysis of policy discourse, as well as of the narratives of people who have witnessed these changes while serving prison sentences.

According to Foucault, ‘one should recall that the movement for reforming the prisons, for controlling their functioning is not a recent phenomenon. It does not even seem to have originated in a recognition of failure. Prison “reform” is virtually contemporary with the prison itself: it constitutes, as it were, its programme’ (1979, 234). Following this logic, our focus on penal reforms that are branded by policy-makers as ‘Europeanizing’ transformations allows us to uncover the pillars upon which contemporary understandings of European punishment stand. Many studies, especially in the fields of international relations, public policy, and European studies, focus on ‘Europeanization’ as the influence of European Union legislation on the national systems of EU member states (Featherstone and Radaelli 2003; Lavenex 2007). In both policy-making and academia, these discussions are often endowed with normative understandings of European values and adherence to ideas and practices of democracy. Yet ‘Europeanization’, in a broader cultural and political sense, is also built around an imagined ‘other’, which, in turn, involves the exclusion of certain groups and create ruptures in the social fabrics of ‘Europeanizing’ states through top-down standardization.

In the former socialist countries of Central and Eastern Europe, ‘Europeanization’ at the political and economic, but also cultural and moral level, has taken up multifaceted and often contradictory meanings, in the context of policy-making as well as public opinion. On one hand, supranational institutions and local political parties, civil society organizations and intellectuals have framed access to the EU as a desirable and necessary step away from their socialist past and from Russia’s sphere of influence. In this discourse, EU access becomes a crucial condition for these countries to develop sound political, economic and social institutions that would grant further integration into the global system. On the other hand, the benefits brought about by membership or closer partnership with the EU and institutions such as the Council of Europe are underpinned by developmentalist approaches that relegate new or prospective members from ‘the East’ to a position of inferiority. While these countries are expected to catch up with Western Europe, the alleged gap between them cannot be filled due to the ways in which the dichotomy of ‘East’ vs ‘West’ is constructed in this discourse. Several studies analyze the permanent condition of backwardness that Western Europe and European institutions bestow upon their eastern counterparts as a feature of Europe’s (post) colonial nature (Dzenovska 2013; Lewicki 2020; Obad 2008). At the same time, the creation of race, gender and class hierarchies unfolding from this approach fosters skepticism and resistance against perceived ‘European values’, such as liberalism and secularism, among those locked into a chronically inferior position. These dynamics do not concern only Eastern European societies *vis-à-vis* the West, but also individuals and groups within these societies who – mainly because of their lower economic and cultural capital – are less suited to ‘becoming European’ (Buchowski 2006; Keinz and Lewicki 2019; Lewicki 2020).

Drawing on critical literature on Europeanization, we focus on two countries from the former Soviet space: Estonia, an EU member-state since 2004, and Georgia, which is not a member but signed an association agreement with the EU in 2016. The logic of country selection is based on a most similar case comparison, with differences in trajectories of prison reform: Estonia reformed and ‘Europeanized’ the prison system it inherited from the Soviet Union more drastically than Georgia. Despite this major difference, we found the narratives among policy-makers and prisoners in the two countries to be similar. Both countries are members of the Council of Europe. Since their independence, Estonia and Georgia have framed their political and economic paths to sever ties with the Soviet past and move towards the ‘European family’, to which they assert to belong. Both countries are considered successful cases of post-socialist ‘transition’. In 2020 the UN Human Development Index (HDI), Estonia is ranked 29^th^, the third highest among post-socialist countries from Central and Eastern Europe (following Slovenia and the Czech Republic).^1^ Post-Soviet Georgia, particularly under Mikheil Saak’ashvili’s presidency (2003–2013), has been depicted by Western political classes and international media as the most ‘European’ country of the region (excluding the Baltic EU members), due to intense reformative efforts, crackdown on corruption, a business-friendly environment, and hostility towards Russia (King 2004; OCCRP 2012).

In both countries, reform of the criminal justice system has been a pillar of ‘Europeanization’. The main discursive thread underpinning the drafting and implementation of policies has been discarding perceived Soviet legacies (Pop-Eleches and Tucker 2011), and embracing what are understood as core ideas and practices of ‘European punishment’. In this paper, we investigate the ambivalent ways in which the ‘Europeanization of punishment’ has been imagined, narrated, and practiced in Estonia and Georgia. We analyze how these images, narratives, and practices of punishment have been created, passed down, enforced, received, contradicted, or resisted by different actors – the political classes of Estonia and Georgia and prisoners in the two countries. This paper, then, examines the narratives produced by politicians and policymakers who plan and carry out reforms, and also narratives produced by those directly affected by the reforms – namely, prisoners. In this way, we also offer an analysis of how elites work with marginalized groups while engaging in a bid to ‘modernize’ their institutions.

First, we locate our study within the framework of critical approaches to European integration and Europeanization. Second, we analyze images, narratives, and practices of ‘Europeanization of punishment’ in Estonia and Georgia. We unpack the discursive construction of Soviet legacies of punishment and the ways in which ‘Europeanizing’ prison reforms are shaped alongside the imperative of moving away from these legacies. Approaching images of ‘Europe’ as an ideational structure discursively produced, we focus on official documents or policy-makers’ statements in which certain concepts and norms are linked to or pitted against ideas of Europe and Europeanness. Empirically, we conduct a critical discourse analysis of official documents that mention Europe, the Council of Europe (CoE), the EU as reference points, or are produced by governments as communication with EU bodies (such as national government responses to reports issued by the European Committee for the Prevention of Torture, CPT). We also draw on ethnographic interviews with former prisoners, which show how prison reforms implemented to bring national penal policies in line with European norms are experienced at the everyday level. We thus bridge policymaking with everyday experiences of those policies.

To bridge these two levels, we conducted data collection and analysis in two steps. First, to analyze Estonia and Georgia’s prison policy discourses, we turned to official documents. These sources include governments’ official responses to CPT reports, politicians’ and policy-makers’ speeches (for both countries); national Prison Services yearbooks and notes from a meeting with two Ministry of Justice representatives (for Estonia); recorded interviews with representatives of the Ministry of Justice and the Special Penitentiary Service (for Georgia). Second, to analyze the narratives of those affected by the policies, we draw on ethnographic interviews conducted in 2020–2021 with people who have served prison sentences in Estonia and in Georgia from the early 1990s to the present time. The interviews, which lasted from 40 minutes to over 3.5 hours, included questions about prisoners’ biographies, prison life and experiences of reforms. In the case of Estonia, our 16 interviews were conducted with former prisoners in halfway houses, while our 36 respondents in Georgia were part of the population of two villages where the research was conducted.The approach to Europeanization as modernization presented in the sections that follow is novel when it comes to prisons and prison reform in Central and Eastern Europe. Contributions on the topic itself are limited, and tackle issues such as the transformation of prison space (Moran 2013; Piacentini and Slade 2015), criminal subculture and prison informal governance (K’ek’oshvili and Slade 2020; Slade and Azbel 2020; Varese 1998), gender and prison (Moran, Pallot and Piacentini 2011; Pallot and Katz 2017), multiculturalism of prison (Curro, Pallot and Zeveleva 2022; Pallot and Zeveleva 2023; Zeveleva 2023), among others. Studies of Europeanization, in turn, as we show below, have rarely focused on punishment and detention. Most importantly, our paper transcends commonly used approaches in the study of Europeanization (which are often based on analysis of discourses produced by elites) by turning to narratives produced by some of the most marginalized members of society – prisoners – who are directly affected by modernizing reforms. We take this approach not only to provide a multifaceted picture of prison reforms in CEE in the framework of Europeanization, but also because we believe that giving voice to prisoners’ perspectives has strong epistemological value. Looking at reforms from a prisoner’s point of view sheds light on the way these policies – and the ideas that underpin them – are understood by people whose practices and relationships are directly shaped by them.

## Europeanization as modernity

We locate our paper within debates on Europeanization as *modernization*, with particular focus on works that analyze dynamics underpinning Eastern Europe’s ‘integration’ in the Western part of the continent. We contribute to this scholarship from the perspective of punishment, analyzing how Europeanization of punishment is framed as modernization of punishment. We investigate contradictions upon which such framing rests, unpacking how images and practices of Europeanization of punishment are promoted, internalized, mistrusted, and challenged at various levels, from policy-making to public opinion and to – the experiences of those most affected, i.e. prisoners. The analysis of Europeanization in the framework of modernization is central to postcolonial and decolonial approaches (Chakrabarty 2000; Mignolo and Walsh 2018; Quijano 2000; Tlostanova 2017, 2019), which pose the nature of colonial history and European modernity as mutually constitutive (Dzenovska 2013). Much of this literature – notably decolonial scholarship – puts at the centre of enquiry the notion of coloniality. Attributing the term to the work of the Peruvian scholar Quijano, Dzenovska suggests that ‘European modernity is constituted through colonial power relations and the associated racial epistemologies of racial hierarchies which emerged in the 16^th^ century with the establishment of the Atlantic trade circuit’ (396). Decolonial thinkers such as Mignolo and Tlostanova state that, unlike colonialism and postcolonialism, which are historically situated, coloniality is a global condition stemming from the colonial system of thought, politics, economics and worldview (2009). Global coloniality is ‘manifested in particular local forms and conditions, remaining, at the same time, a connecting thread for the understanding of dissociated forms of modernity’ (Tlostanova 2019, 166). Decolonial approaches tackle at multiple local levels ‘the long-lasting ontological, epistemic, and axiological traces left after any colonialism seems to be a matter of the past’ (166). As an enduring global condition, coloniality ‘shapes the contemporary political topography of Europe, where it has been crucial in maintaining a distinction between […] “mature” and “less mature” political subjects’ (Dzenovska 2013, 396). In the context of post-socialist Europe’s ‘integration’ into its Western liberal side, Eastern European post-socialist countries are given – to different degrees – the status of ‘almost-but-not-quite- European’. These societies’ level of Europeanness can be increased (without, however, being fully accomplished) to the extent to which Eastern European countries embrace Western Europe’s more developed system of values and practices (Dzenovska 2013). It is in this developmentalist light, which poses Western modernity as the most desirable (if not the only possible) system to aspire to, that we understand ‘Europeanization’ and its constitutive ideas, narratives, and practices.

The term ‘“Europeanization”’ is not applied uniformly in the literature (Börzel 1999; Checkel 2001; Olsen 2002). Olsen, for example, refers to it as the process of ‘“adapting national and sub-national systems of governance to a European political centre and European-wide norms”’, which includes engagement with both EU official institutions and other bodies like the CoE (2002, 924).^2^ However, in addition to policy implications of EU enlargement as a bureaucratic entity, and alongside the ‘“political and economic reality of Europe as a place”’, these processes are connected to a *discourse* of Europe and to ‘“Europe as an idea and an ideal”’ (Kuus 2005, 567).The relevance of moral and cultural arguments is particularly evident in the EU’s expansion eastward, which rests on narratives of a continent finally united and free.^3^ The discourse on Europe, as all discursive formations, has a normative function which establishes the features that a ‘good society’ must have, and sanctions or excludes deviations from these features.

Adamovsky (2005) focuses on how this normative function has constructed Eastern Europe as the Other *vis-à-vis* ‘Europe’ proper (that is, Western Europe), and, referring to Said’s work (1979), defines the discursive formation of the West on the East as Euro-Orientalism. Analyzing its origins and evolution, and how it has been actively shaped and spread by academic institutions, Adamovsky defines Euro-Orientalism as the means by which ‘the West symbolically organizes and regulates its relationships with the area of the world called Eastern Europe’ (609). These relationships are framed around the image of Eastern Europe, and Russia in particular, as a ‘land of absence’, whereby the region is defined through what it is *not* and what it lacks (591). While these supposedly missing features varied across different historical periods, the main narrative thread which has underpinned the relationship between ‘Western’ and ‘Eastern’ Europe is one of modernity *vis-à-vis* the lack of it – or, in the case of capitalist democracies vs socialism, the ‘right’ type of modernity against a ‘wrong’ one. As the foundational discourse for Europe’s definition of itself ‘“through continuous (post) colonial and (post) imperial entanglements between *Europe* and *non-Europe*”’ (Lewicki 2020, 129), Euro-Orientalism has put Eastern Europe in a position of chronic inferiority. These hierarchies of political, economic, cultural, and moral standing have often been internalized by Eastern European political classes and publics (Obad 2008). The ‘“catch-up logic of development”’ that accompanies this sense of inferiority frames Western-type modernity as unattainable (Rekhviashvili and Sgibnev 2021).

These debates have become increasingly relevant in the context of the enlargement of the EU and other Western institutions, such as NATO. Prospective new members’ inclusion is evaluated against these societies’ degree of political and economic ‘otherness’ *vis-à-vis* the West, and their potential to close this gap – which, is un-closable by definition. Such evaluation defines ‘to what extent their [these countries’] affairs can be left in their own hands and how much they can be helped, advised, or forced to become something else’ (Adamovsky 2005, 619). In this regard, critical literature from across the social sciences has analyzed the impact of reforms pushed through by European agencies and other international institutions, and uncritically implemented by local governments, on Eastern European livelihoods, landscapes and infrastructures (Klumbyte 2009; Mincyte 2014; Rekhviashvili and Sgibnev 2021; Resnick 2018). Our analysis of prisoners’ narratives in Estonia and Georgia follows this ethnographic approach, which investigates how supposed ‘European values’ translate into reformative processes, and are in turn experienced by ordinary people – theoretically the targets, and the main beneficiaries, of these policies – in their everyday life.

At a first glance, narratives and practices analyzed in these studies frame the elimination of features associated with the socialist past as a precondition for achieving modernity. To facilitate this transition, Western institutions deploy ‘systematic guidelines and cadres for the reforms, […], international institutions, experts, nongovernmental organizations, and companies’ (Adamovsky 2005, 622). However, while hostile feelings against the socialist past – and, for many countries, Russia’s ongoing perceived threat – are common across Central and Eastern European political classes and populations, the acceptance and upholding of Western Europe’s purported values does not go unchallenged. In his ethnography of Polish EU civil servants in Brussels, Lewicki (2020) explores moral and cultural hierarchies established by the compliance of EU bureaucrats’ bodily performances with what he defines as ‘Eurostyle’. Distinctions drawn by these racialized hierarchies among Polish EU civil servants, he argues, foster challenges against ‘Eurostyle’ through the performance of a conservative, religious Polishness, which, in turn, reflects skepticism towards cosmopolitan liberal values that are allegedly part of the EU’s collective identity (128–129). Kuus (2002) highlights the uneasy coexistence of Estonian narratives when the country was pursuing membership in the EU and NATO. Political, academic and popular discourses favoured international integration as a way to protect Estonian identity and sovereignty from Russia’s threat. At the same time, international institutions’ pressure on Estonia to naturalize their Russian-speaking population, which is depicted as representative of this threat, framed this integration as dangerous for national identity. In Rekhviashvili and Sgibnev’s analysis of infrastructural development in the post-Soviet space (Rekhviashvili and Sgibnev 2021), the imperative to catch up with the unreachable Western-type modernity is articulated against the background of loss of socialist modernity. The tense intertwinement of inferiority *vis-à-vis* the West as the sought future and inferiority *vis-à-vis* the past is mobilized by political classes to put forward problematic infrastructural projects across the region – such as hydropower plants, or disinvestment from publicly run transport services in the South Caucasus and Central Asia.

Policy makers and public discourse in Estonia and Georgia have framed post-Soviet prison reforms as a modernizing attempt to move away from unwanted legacies. Integrating the literature analyzed above with our empirical findings, we discuss how these legacies are not uniformly understood as negative legacies of the Soviet past. We investigate the conflicting and ambivalent elements underpinning images and practices of ‘European punishment’ and ‘Europeanization of punishment’ from different perspectives. Our analysis focuses on the discrepancies between benefits of reforms promoted by EU institutions and local governments and prisoners’ living experiences. We also highlight that contradictory attitudes emerge not only between policy-makers and prisoners, but also within the policy-making sphere and within prisons. The paper explores how images and practices of ‘modernization’ and ‘humanization’ of prison are appropriated by local policy makers – and often by the media and the public as well – to demonstrate belonging to Europe through competently implementing ‘European punishment’. However, we argue that images and practices of ‘European-like’ prison reforms are mobilized by local leadership to pursue internal political goals. References of ‘Sovietness’ as indisputably negative may be strategically attached to internal ‘others’: a deprived region in the north-east and the Russian-speaking minority in Estonia, and political predecessors and opponents in Georgia. Yet, understandings of ‘Sovietness’ vary according to the political projects pursued in each country. These projects may be problematic inasmuch as they contradict the same ‘European values’ and regulations which they claim to reflect. The ambivalence underpinning policy-makers’ images and practices of ‘European punishment’ are reflected in prisoners’ lived experiences of prison reform, and replicated by certain aspects of the political, economic, legal and cultural relationships between EU institutions and ‘reforming’ countries. ‘Europeanization’ as an idea, a political project and a set of practices is at the same time endorsed, exploited and challenged by multiple actors, revealing the contradictory nature of relationships between the ‘centre’ and ‘periphery’.

## Soviet ‘legacies’ and the Europeanization of punishment

Drawing on the assumption that penal systems are indicators of a country’s level of development, the CoE promotes its vision of human rights through the European Prison Rules, and by recommending reforms that bring national penal policies in line with these principles. This approach does not problematize the idea of punishment, nor does it bring into focus broader social processes that provide the backdrop for crime rates, namely unemployment and poverty. Instead, it reproduces the idea that punishment must be present in society in the form of prisons, and these prisons should be humane. Likewise, bringing national penal systems up to ‘European standards’ is considered a priority in the political agenda of countries that have recently joined, or aspire to join, Western international institutions.

General perceptions of European punishment echo narratives of modernity – and, therefore, inherent superiority – attached to what is considered to be (Western) European culture, society and politics. The main attributes of ‘modern punishment’ are efficiency, transparency and respect for prisoners’ rights. European punishment, therefore, is humane punishment. Such qualities are enshrined in the European Prison Rules, issued by the CoE in 2006. Their principles focus on a fair approach to prisoners, including the prohibition of ‘torture and inhuman or degrading treatment’, as well as the duty to provide prisoners with a healthy environment, which is conducive to incarcerated people’s rehabilitation and re-entry into society as the ultimate goal of punishment. The Prison Rules stress the individuality of every prisoner, whereby the punishment and rehabilitation path should be planned in line with a person’s unique mental, physical and social situation. Each prisoner should be given a choice among a range of meaningful activities available in custodial settings, from work to education, to arts and sports. The Prison Rules also include clauses about discrimination: fairness should come with the recognition of each prisoner’s individual needs (Council of Europe 2006; Mulgrew 2016).

Central and Eastern European countries have largely framed prison reforms as overcoming perceived socialist legacies. These legacies are depicted in contemporary policy discourse as the repressive nature of the socialist punitive apparatuses, with arbitrary arrests, unfair trials, disproportionate sentences and appalling conditions of detention. As a prison social worker stated in an interview, after the end of socialism these countries’ prison systems ‘started following the Western model to become more humane’.^4^ Such ‘humanization’ of punishment entailed the partial or total replacement of military personnel with civil workers among prison staff, and the shift from discipline and re-education through labour to rehabilitation through individual sentence plans. In Estonia and Georgia, reforms have also focused on prison space and governance. Moran and Jewkes (2015) argue that prison architecture is a material expression of the goals of a criminal justice system. Different ‘penal philosophies’ have conceptualized prison space along the continuum between collective and cellular arrangements. In the history of prison space, these two approaches are linked to principles and practices of Soviet and Western-style penal systems respectively. Focusing on the former, Piacentini and Slade (2015) highlight the impact of collectivism on how punishment was conceived in the Soviet Union, which determined the spatial organization of Soviet prisons. Prisoners were accommodated in *otryady*, that is, barracks or detachment blocks of 2–3 floor buildings made up of mass dormitories, where they lived openly and communally. Drawing on Kharkhordin’s work on the Soviet/Russian concept of *kollektiv* (Kharkhordin 1999), the authors argue that, together with mutual surveillance, a key feature of collective organization of prison space is self-governance. In Soviet penal institutions, prisoners had positions of formal authority in various organizations and self-organized councils. Along with these formal arrangements, hierarchies of prisoners headed by so-called ‘thieves-in-law’ – in Russian *vory-v-zakone* - developed with the more or less open consent of the administration for a parallel, informal oversight of discipline and conflict resolution. The ‘thieves-in-law’ are also representative of a prison subculture whose moral and cultural power inside prisons, as well as across society outside, has facilitated the criminal world’s grip on various local economies and politics, particularly in the last years of the Soviet regime and throughout the 1990s (Galeotti 2018; Varese 1998).

Post-Soviet reforms focused on the spatial re-ordering of prisons from large collective spaces towards cellular structures. Both local governments and international institutions framed these changes primarily as a human rights concern to alleviate the ‘pains of imprisonments’ (Sykes [1958] 2021) caused by lack of privacy. The atomization of prison space also aimed to break prisoner hierarchies and formalize prison governance. Along with the struggle against prison subculture, these efforts have focused on taking power away from prisoners and situating it in the penal administration’s hands. Reformed penal orders leave to prisoners little initiative and decision-making power on the everyday running of the prison, which relies also on the external expertise of professionals such as psychologists and social workers.

In the sections below, we turn to the question of how penal reforms in Estonia and Georgia in the last three decades have been framed around ideas of ‘European punishment’ by governments.We focus on these countries’ discourses around reforms of prison space and prison governance, arguing that in both cases, albeit differently, local policy-makers bring about images of modernization and humanization of the prison system away from Soviet legacies strategically and performatively. While used by local governments to show European and transnational institutions that they are up to international expectations, these images also convey discourses and practices which may be in contradiction with perceived ‘European values’ and the ‘humanization of prison’. These tensions are reflected in practices of punishment, which are embodied in the effects of penal reform on those who are incarcerated. To analyze prisoners’ perceptions we draw on qualitative interviews conducted in Estonia and in Georgia. In this way, we analyze the impact of Europeanizing and modernizing discourses at various levels, from policy-making to practices on the ground in relation to prisoners. We also focus on the role that European institutions play in producing such contradictions, highlighting the violence inherent to hierarchical relations that discourses of ‘Europeanization as modernization’ establish between ‘Europeanizing’ agents and ‘to-be-Europeanized’ subjects.

## Estonian and Georgian penal reform in policy discourse

Estonia began its prison reforms in the early 1990s, shortly after gaining independence, and the policy documents produced thereafter include two major reference points: the country’s Soviet past, which is depicted as contradictory to both European and Estonian values surrounding punishment; and Estonia’s internal ‘other’ as the Ida-Viru county, a north-eastern Russian-speaking region. These discourses intertwine to marginalize the country’s Russian-speaking minority. The analyzed documents argue that Soviet legacies have made some elements of the penal system difficult to improve. For example, the high number of prisoners in Estonian prison system is linked to ‘the decomposition of the village society due to the acts of the occupant regime and repressive penal system which was remote from European values’.^5^ Soviet legacies clash with ‘specifically Estonian’ prison policy, notably with the use of Estonian language in prison. Since the penal system was controlled by the USSR, ‘it became impossible to pursue a specifically Estonian effort to rehabilitate inmates, in particular as 95–91% of the staff did not speak any Estonian’.^6^ Both Estonia and Europe emerge as entities that embody values and policy orientations antithetical to the Soviet past.

Prison space is another manifestation of Soviet influence on the Estonian system. The barrack-type communal living setup of Soviet correctional colonies ‘make it relatively difficult to achieve the goals of imprisonment – to bring the person back to the society as a law-abiding citizen’.^7^ Policy discourse also links Soviet legacies to prisoners’ informal governance:

Criminals were appointed as foremen and ‘authority men’ and in return for securing ‘order’ and production their violations of the basic rights of the detainees were often condoned. This developed a negative attitude among inmates towards the law and the regime brought with it a growth of recidivist crime in the middle of the eighties.^8^

Official discourse considers the re-building of Soviet-era correctional colonies into cell-based prisons as crucial for battling these legacies and upholding European Prison Rules. Indeed, Estonia implemented these changes: while in 2002 there were 10 prisons in the country, by 2020 the number was reduced to three new penal facilities, one of which was built in Ida-Viru county.

Ida-Viru is a geographical and administrative, but also a cultural and linguistic reference point. The Estonian Census estimates that over 73% of the population of Ida-Viru self-identify as ethnic Russians. About one-third of Estonia’s entire Russian-speaking minority (372 480 people, or 29.6% of the population)^9^ live in this county, where poverty and unemployment are more widespread compared with other regions (Statistics Estonia n.d).^10^ This adds a strong ethnic dimension to incarceration, which implies that reform discourses disproportionately marginalize Russian-speakers. A quote from the Justice Minister’s speech at a conference of CoE Prison Directors, hosted in 2018 by Estonia in Ida-Viru county, reflects these dynamics:

The geographical location of the Department of Prisons of the Ministry of Justice – Jõhvi – is not accidental, nor based on economic calculations. The eastern border region was the most intensively Sovietized area of Estonia in the aftermath of the Second World War … Thus, the political agenda of moving one of the three prisons of Estonia, Viru Prison, and the Prison Department … to Jõhvi, has been the integration of the eastern region of the country.^11^

Referring to the move of penal institutions to Ida-Viru, the 2015 Prison Services yearbook criticizes the ‘many sceptics’ who ‘just could not believe that it was possible to form a national language speaking, nationally minded body of officers here, to create a modern custodial institution out of thin air’.^12^ Here too, this ‘eastern border’ region is depicted as something that many locals allegedly do not perceive as part of Estonia:

Not that the people here don’t think of Narva or Sillamäe as part of our country, but the identity of Ida-Viru County as their home is perceived differently in many ways. It is like Estonia proper started only west of Padaorg Valley, a place where locals hardly venture to, and from where only visitors and tourists come. The vision harks back to the Soviet times and is very difficult to get rid of.^13^

Estonia’s regional internal ‘other’ overlaps with its historical ‘other’ in these discourses. Soviet legacies are incompatible with European and Estonian prison policy efforts and hamper reforms. The lingering of these legacies ascribed to Ida-Viru creates an image of this county as problematically separated from the rest of Estonia. Yet, the analyzed documents argue against this image and the ‘skeptics’ who voice stigmatizing stereotypes of Ida-Viru, stating that the region can successfully host a large state institution. At the same time, such institution is a site of detention tasked with carrying out punishment, within which Russian-speakers, and especially stateless Russian-speakers (or holders of so-called ‘gray passports’), are overrepresented.^14^ The Russian-speaking region is stigmatized discursively, while Russian-speaking population’s marginalization is also exacerbated by Estonia’s criminal justice system.

Estonian authorities depict the construction of the prison as an important way to make the government more visible in the region. The Governor of Joehvi even pointed out that ‘the role of promoting active young people’s flow into the region is fulfilled by an unexpected institution in ordinary people’s eyes: a prison’.^15^ The aim to attract ‘a national language speaking, nationally minded body of officers’ was based on the idea that prison staff would come from other regions, and that Estonian language requirements for the job would be stringent. This trend was confirmed by policies launched in 2010, which stipulated that fluency in Estonian would become mandatory for prison staff jobs. In combination with the new cell-based prison architecture, this shift towards Estonian-speaking staff led to significant formalization of prisoner-staff relations in Estonia’s new prisons. We discuss the reception of this change in the sections below.

Prison reforms in Georgia, too, have been at the forefront of discourses and practices of social, political and economic ‘modernization’. Similarly to Estonia, predominant political narratives have framed ‘modernization’ as moving away from the past towards (re-)integration with the West (Curro 2023; Kakachia, Lebanidze, and Dubovyk 2019; Rekhviashvili and Sgibnev 2021). In particular, the government led by Saak’ashvili, which came to power after ousting Shevardnadze with the so-called Rose Revolution in 2003, was characterized by a strong pro-West and anti-Russia discourse (Kakachia, Lebanidze, and Dubovyk 2019). The government shaped criminal justice and prison reforms around a zero-tolerance model to crack down on lawlessness and informality, which were seen as main causes of Georgia’s political and economic underdevelopment (Curro 2015; Rekhviashvili 2015). As a result, by the end of 2000s Georgia became one of the biggest incarcerators worldwide (K’up’at’adze and Slade 2014). Moreover, police and prison personnel’s arbitrary use of power became widespread (Slade 2012).

The government justified this harsh approach with the need to reassert state power over a society which, in Saak’ashvili’s words, ‘was ruled not by (previous president) Shevardnadze, but by “thieves-in-law” and bandits … ’^16^ The thieves’ normative power across Georgian society consolidated in, and spread from, national prisons, favoured by collective spatial arrangements. The government aimed at breaking thieves’ power by separating powerful criminal figures across different prisons and imposing harsh detention conditions. The same stringency was applied outside the prison to trump the criminal world’s influence on society and culture with loyalty to the state (Slade 2007). Saak’ashvili framed this approach as it follows:

We should put everyone in jail in accordance with the law and we should amend the criminal procedure code so that no one can be released through conditional sentences … Let everyone – criminals and their political and other kinds of supporters – know that whatever they do, we will anyway establish order … our streets will be cleansed of this criminal rubbish through the protection of all laws and principles of democracy.^17^

Post-Rose Revolution Georgian discourse on crime and punishment defines Soviet legacies primarily by lawlessness and chaos, which endured the USSR collapse and tainted the country’s politics and economics throughout the 1990s. The reassertion of state power envisaged by Saak’ashvili’s government to discard these legacies was not always compatible with democratic values and human rights (Mitchell 2009). The government was ambivalent towards supranational institutions’ evaluations and recommendations on its prison management. In responses to reports, government representatives assertively rebut the CPT’s observations, identifying the delegates’ poor understanding of Georgian specifics as a main cause for their – misplaced – criticism. Here is the response to a CPT recommendation to increase prisoners’ living space:

The limited financial resources of the state do not allow us to increase the living space at once, however we will be guided by … the new Code on Imprisonment of Georgia, which provides: ‘Authorized state institutions shall take all possible measures to **gradually** increase the living space per prisoner … , in order to comply with International Standards, however considering state possibilities’.^18^

While European Prison Rules state that ‘prison conditions that infringe prisoners’ human rights are not justified by lack of resources’ (CoE 2006, 7), the government’s response prioritizes Georgian norms over international standards. Responses also gloss over prisoner ill-treatment allegations mentioned in reports, asking the CPT to provide more evidence of such cases to have proper investigations, or denying allegations altogether.^19^ Here, the attitude towards crime and punishment differs from the CoE’s approach to the deprivation of liberty as ‘a measure of last resort’ (CoE 2006, 5) and the focus on prisoners’ rights and rehabilitation. Modernization discourses promote the country’s Westernizing look to the outside – Western governments, international institutions, media – while also boosting internal plans for consolidating statehood that may be at odds with perceived ‘European values’. The Soviet past is despised less for its oppressive nature than for its legacy of lawlessness, which challenges the government’s ability to control society, politics and economics.

Alleged abuses under Saak’ashvili’s government became public few days before the 2012 elections, when TV channels aired recordings of guards’ violence against inmates in the prison of Gldani, Tbilisi. The resulting scandal contributed to defeat the incumbent leadership, which was already unpopular for increasing inequality and authoritarianism. The new government, led by the Georgian Dream (GD) party, declared a mass amnesty to break politically and symbolically with its predecessors. Reducing the high number of prisoners, the amnesty was part of reforms that government and media presented as fixing past wrongdoings with a full-scale adoption of European prison standards. The legacies against which the Europeanization process is pitted refer no longer to the Soviet past, but to the previous government. The contrast with the former leadership is expressed in the response to a report issued after the CPT’s first periodic visit under the GD government:

As you are aware since coming to power in October 2012, the new government had to deal with numerous grave concerns, including a widespread and systemic problem of torture and ill treatment in the Georgian Penitentiary system. Decisive steps were taken in a short period of time to bring the penitentiary system closer to the requirements of international human rights standards. The comprehensive reforms initiated by the Ministry of Corrections aim at … gradually introducing a modern prison management methodology, based on individual approach, risk assessment and sentence planning.^20^

Here the modernity of prison management is explicitly linked to the European system. Policy-makers show a detailed awareness of what this ‘modernity’ means in concrete practices and regulations. The faithful reproduction of the CoE’s language aims to provide evidence of cultural competence within the European institutional framework. Georgian policy-makers’ performative use of official language reminds of Lewicki’s ‘Eurostyle’ (Lewicki 2020) and the hierarchies established by the ability, or inability, of conforming to it.

Analyzing the language used by two governments to present their crime and punishment policies to internal and external audiences (National Security Council, CPT, national and international media, foreign experts) highlights important differences. Under Saak’ashvili, prison reforms were part of an assertive project of state building. Internally, this project was supported by a grandiose rhetoric based on the opposition between the backwardness of the Soviet era and the 1990s and the modernity of the post-Rose Revolution leadership’s political view (Curro 2023; Gotfredsen 2014). While this discourse boosted Georgia’s (re-)integration into the European/Western family also with international institutions and media, the government treated external criticism on its project as untimely and misconceived interference. The GD government’s^21^ use of language reproduces faithfully the CoE terminology to present their prison reforms as conform to ‘European standards’, which would indicate Georgia’s entitlement to belong to the modern West more broadly. Following this discourse, while Saak’ashvili’s government presented itself as ‘European’ but disregarded procedural fairness and human rights in the penal system, their successors are overturning this legacy by truly committing to European modernity. Despite these differences, however, both governments’ languages and attitudes towards EU institutions underpin strategies boosting Georgia’s position on the international scene as a way to legitimate externally the pursuing of internal political projects.

In the following section, we analyze the contradictory narratives of those who experience the outcome of such political projects at the everyday level: prisoners who have spent time in Estonian and Georgian prisons since the end of the Soviet Union. There are crucial differences between the two countries, notably when it comes to the extent to which prison reforms have been implemented and the ‘success’ in achieving the declared goals of these reformative efforts. However, as it will emerge from the investigation of prisoners’ stories, the two cases also share some common narrative threads, particularly when it comes to compare life in a system inherited from Soviet times with conditions brought about by post-Soviet reforms. The perceptions and feelings expressed regarding the organization of space, the relationship with prison staff and prisoners’ power in the organization of prison life resonate in similar ways across the stories of both Estonian and Georgian (former) prisoners. Keeping these similarities in mind is important for a critical analysis of the implementation of ‘European’ reforms, whose appropriateness and inherent superiority *vis-à-vis* other systems is often uncritically taken for granted both by those who ideate and promote these reforms and those who endorse them.

## Practices of punishment in prisoner narratives

Narratives of prisoners who have experienced prison reforms in the last 30 years echo and refract the contradictions we identify in policy-makers’ discourse on the Europeanization of punishment. This section thus turns to our analysis of interviews we conducted with former prisoners. In the case of Estonia, movement to cellular structures and Estonianization of prison staff – which political classes portray as the pillars of Europeanizing reforms – created feelings of alienation and loss of control over activities inside the prison among incarcerated people. Similar narratives emerge from interviews with Georgian prisoners, who compare social relationships, solidarity and reciprocity of past prison life to a present in which prisoners are isolated and denied agency and respect. Prisoners also argue that in some aspects the reformed prison systems became more brutal.

The prison reforms discussed above firstly led to the formalization of prison governance, which entails moving control from prisoners’ informal hierarchies into prison staff’s hands. One former prisoner in Estonia described the transformation in the following way, highlighting that the new order required a staff turnover:

They’re replacing the old guards with new guards, youth. They figured that these old police guys, the old guard, do a lot of stuff the new ones don’t do. Because the old ones were used to having good relations with prisoners, [but] the administration wants rigidity, so that the guards have distance above the prisoner … He’s police, you’re a prisoner.^22^

Some participants also point out that the distance between Russian-speaking prisoners (who are overrepresented in the Estonian system) and prison staff was exacerbated along ethno-linguistic divides by the reform that introduced mandatory fluency in the Estonian language for prison staff.

In Georgia, the power of informal hierarchies and criminal subculture has been an ongoing problem which prison reforms have approached in different ways and with various outcomes (K’ek’oshvili and Slade 2020; Kukhianidze 2009; Slade 2016). From policy-makers’ perspectives, prison informal governance is a negative phenomenon not only because it takes power away from the administration and the state, but also because it is a source of prisoner-to-staff and prisoner-to-prisoner violence.^23^ Many former prisoners, however, connect the formalization of prison governance with the loss of community and everyday solidarity among inmates. The following quote compares prison life before and after the state cracked down on prisoners’ informal power and connections, often resorting to non-transparent and possibly violent methods:

I told you that those years, it was better, right? 2003 and 2007 … you are a prisoner, you still had your freedom. … Everyone had a sweet relationship with each other, … here we were taking care of each other … That’s how normal and peaceful life was. Life in prison was not difficult for anyone. Even if you did not have a patron from the outside, the patron would appear inside.^24^

The degree and kind of loss varied according to a prisoner’s position within informal hierarchies. For top members such as thieves-in-law, reforms implied the dissolution of their power in prison through isolation and harsh detention conditions. For prisoners close to criminal authorities, the eradication of informal governance meant a loss of material and non-material privileges, as well as increasing vulnerability *vis-à-vis* criminal justice for their alleged support of criminal subculture.^25^ Less powerful prisoners, however, could have arguably welcomed the formalization of power in prison as a less arbitrary, more impartial and attentive form of control. Yet, along with reduced sociality and more strictly disciplined human interactions, former prisoners’ narratives from Georgia and Estonia associate with these reforms the suppression of their agency and autonomy, rather than a gain in their own safety and a fairer system of governance. These feelings were widespread among our research participants, which is partly related to the sample we have worked with. Former prisoners we interviewed in Estonia and Georgia were generally well acquainted with, and often involved in, prison subculture, although in most cases they did not hold a position of high authority within the informal hierarchy.^26^

The move from communal life in Soviet-style barracks to cell accommodation has been a prominent intervention associated with the formalization of governance in the framework of prison reforms. In Estonia’s new prisons, the transformation of space, which entailed the removal of nearly all common areas, was accompanied by a shift in scheduling. Prisoners are now allowed outdoors for one hour a day, which, in some prisons, involves taking walks in a special area on the roof of the prison. This is a major change from pre-reform prisons was received negatively, and prisoners nostalgically recalled a time when they could ‘leave at 6 in the morning … and just be outside till 10 in the evening’, as one study participant recalls. This former prisoners also recounted how in an old, pre-reform prison of Estonia he had arranged for fertile soil to be delivered to the prison in order to create a garden, where he grew his own vegetables and would eat them during his meals. He praised the nutritional value of the fresh vegetables, the sensation of working with your hands and producing something within the grounds of the prison.^27^

Georgian former prisoners similarly stress the ‘normality’ of past prison life, in which incarcerated people had a certain degree of freedom of movement, socialization and activities. This relatively carefree condition, in which prisoners could also acquire new skills, is expressed by one participant who served time before the Rose Revolution, in 1999–2001:

In the cell we had a stove, we cooked good food. We played nardi (backgammon) and so on. We had nothing to do and no worries. […] Life in prison was nothing in particular, it was just like normal life. The only thing from morning to evening was what to eat and what to do. And we made the most delicious food! I learnt how to make food in prison.^28^

Penal sociologists and other scholars have discussed the ambivalent ways in which prisoners may perceive the ‘tightness’ of prison control and disciplinary power (Alford 2020; Crewe 2011; Crewe and Ievins 2021; Shammas 2014). The surveillance of incarcerated people is depicted as highly uncomfortable in both academic work and literary fiction. However, the loosening, or absence, of such all-pervasive power can be experienced as institutional neglect (Alford 2000; Crewe and Ievins 2021). Moreover, inmates may be unsettled by the need for self-regulation and individual responsibility that some prison systems bestow upon them (Shammas 2014). In our study, prisoners’ partial control of prison space and activities is associated in prisoner narratives with a more humane dimension of prison life and its social fabric. Policy papers and international organizations’ reports often link informal governance to violence, unjust privileges and recidivism.^29^ Yet our study participants from Estonia and Georgia long for a ‘looser’ type of institutional power, claiming it granted them a higher level of freedom and agency enabled solidarity and reciprocity among inmates, a spontaneous development of skills and creativity, as well as more equal and humane relationships between prisoners and staff. What EU institutions and local policy makers describe as an enhancement of prisoners’ quality of life and perspectives of rehabilitation is framed by inmates themselves more in terms of loss than of gain.

## Conclusions

The pillars of what is understood as ‘European punishment’, enshrined in the Council of Europe Prison Rules, conceives the deprivation of liberty as a last resort. These rules state that, whenever necessary, detention should be focused on prisoners’ physical and psychological wellbeing, with the primary goal of rehabilitating and returning incarcerated people to society as ‘healthier’ individuals. To achieve that, prison management should provide prisoners with quantitatively and qualitatively appropriate living space, a wide range of stimulating activities, and a disciplinary apparatus that respects their rights and does not discriminate them.

In most of Central and Eastern European countries, prison reforms have been at the forefront of transformations following the end of socialist regimes. Both Western institutions and, largely, local political classes have framed such transformations as processes of modernization from the backward socialist order towards (Western) European development standards. According to these narratives, reforming punishment in a ‘European’ fashion means modernizing punishment – that is, making it humane. Prison reforms implemented in Estonia and Georgia since the end of the Soviet Union have aimed, among other things, at reorganizing prison space from collective to cellular arrangements, as well as at tackling the power of informal governance. In local policy-makers’ discourse, these reforms entail a shift towards a more efficient and humane system, with the discarding of Soviet dysfunctional and oppressive legacies as a necessary condition to ‘return’ to the ‘European family’ (Lewicki 2020; Obad 2008). However, in both cases examined here, ideas and practices of ‘Europeanization of punishment’ entail contradictions at multiple levels.

First, among Estonian and Georgian political classes, the language of ‘Europeanization of punishment’ comes across as a device to present these countries’ suitability for modern ‘European standards’ to an external audience of international institutions, media, and publics. At the same time, the local political leadership pursues its own internal political goals, which may be at odds with the same ‘European values’ which these governments declare to take as models. Discrimination against Russian-speaking minorities and their over-incarceration in Estonia, as well as the arbitrary use of power for fighting crime in Georgia are cases in point. Local political discourses deal with these contradictions using various strategies and narratives. While the need and the entitlement to belong to the ‘European family’ is not questioned, mainstream discourses claim a kind of uniqueness derived by the pains and troubles inflicted on these countries’ social, political and economic fabric by Soviet domination. In the name of ‘Europeanization’, Estonian and Georgian political classes advocate for themselves the right of dealing in their own way with the legacies of this domination. The same European community they want to join, they claim, may be unable to understand the unique challenges that these societies have faced and continue to face. Second, as it emerges from prisoners’ narratives in both countries, the key terms of ‘European punishment’ language – ‘individual approach’, ‘sentence planning’, ‘rehabilitation’ – do not necessary translate into better life conditions from prisoners’ perspectives. Prisoners tell contradictory and ambivalent stories about their losses and gains over the course of prison reforms. In our cases, policy-making modelled on EU standards as the most ‘humane’ type of punishment appears to be detached from incarcerated people’s everyday experiences. According to many prisoners, the shift to cellular accommodation and the related disempowerment of informal hierarchies have curtailed a sense of community and agency among inmates and brought about a bureaucratized, impersonal approach with prison staff and administration. While we should not praise conditions of imprisonment under Soviet-type systems (and in the first post-Soviet decade before reforms), which were marked by violence and arbitrariness, the longing for a prison life dictated by less strictly regulated and more direct interactions among prisoners and between prisoners and staff must be taken into account for a critical analysis of reformative processes. Reform is a part of the functioning of the prison itself, and so long as prison is conceived as an institution of punishment, reform programmes are also still centered around notions of punishment, even in the context of moving towards a human-rights-based regime. The question of reform then becomes ‘how can we punish better?’, and the results are bound to have uneven effects throughout the general population, as well as the prison population specifically. In the case of post-Soviet countries aspiring to a Europeanized prison model, we see a hierarchy of ‘backward’ punishment and ‘better’ punishment emerging in policy discourse, but this hierarchy can be refracted, turned upside down, or reinterpreted and challenged when we examine the experiences and narratives of the prisoners who reap the direct effects of prison policies.

Thus, our analysis of images and practices of ‘Europeanization of punishment’ in two post-Soviet societies brings punishment into the growing debate in critical studies of Europe and its (post-)colonial nature. Punishment is not just yet another case study of the relations between the European ‘centre’ and its ‘peripheries’. Images and practices of ‘European punishment’ draw upon core principles which have discursively structured the dichotomy between the democratic, developed West and the (post-)socialist, underdeveloped East: humanness, care for each individual’s value and uniqueness, and, ultimately, freedom. Ideas promoted by the European Prison Rules frame imprisonment as a ‘last resort’ occurring only after fair procedures, and of conditions of detentions which ‘shall approximate as closely as possible the positive aspects of life in the community’ (CoE 2006, 7), are the pillars against which the arbitrariness and brutality of the penal systems in socialist regimes are discursively pitted. Subsequently, the humanness of punishment is the basis upon which the moral, cultural, social and political superiority of the West *vis-à-vis* the East has been built in discourses and practices. A focus on the ambivalent images and practices of ‘Europeanization of punishment’ among local political classes, in prisoners’ narratives, and in European institutions’ policies, casts some light on the ways visions of ‘Europe’ and related hierarchies are created, mobilized, and experienced at multiple levels, as well as on the contradictions and fractures that these visions carry.

## Notes

1.
http://hdr.undp.org/en/content/human-development-index-hdi

2. In the case of Georgia, a more precise definition includes the export of forms of political organization and governance beyond the EU.

3. The EU’s ’Eastern enlargement’ has so far included 10 former socialist countries from Central and Eastern Europe. Czech Republic, Slovakia, Estonia, Lithuania, Latvia, Poland, Hungary and Slovenia joined in 2004, while Romania and Bulgaria joined in 2007.

4. Interview with a prison social worker, Czech Republic, July 2021.

5. Estonian government response to CPT report, 2002, https://rm.coe.int/CoERMPublicCommonSearchServices/DisplayDCTMContent?documentId=090000168069573c. Accessed on 27 June 2021.

6. Estonian government response to CPT report, 2002, https://rm.coe.int/CoERMPublicCommonSearchServices/DisplayDCTMContent?documentId=090000168069573c. Accessed on 27 June 2021.

7. Prison System and Probation Supervision Yearbook, 2004, 26: https://www.vangla.ee/sites/www.vangla.ee/files/elfinder/dokumendid/estonianprisonsystemyearbook2004.pdf.

8. Estonian response to CPT, 2002, 1–2

9. Statistics Estonia, 2020.

10. Labor force participation in Ida-Viru County is 57.6%, compared with 74.8% in Harju County surrounding Tallinn.

11. Opening plenary session at the 2018 Conference of the Council of Europe’s Directors of Prison and Probation Services, hosted by Estonia. Speech by Urmas Reinsalu, Minister of Justice.

12. Prison Service Yearbook 2015, 7: https://issuu.com/vanglateenistus/docs/vanglaameti_aastaraamat_veeb.

13. Ibid.

14. On the ‘gray passport’ system, it institutionalization, and the dynamics of exclusion that it brings about see Cianetti 2019, 41, European Network on Statelessness 2015, 6.

15. Prison Services yearbook 2015, page 12: https://issuu.com/vanglateenistus/docs/vanglaameti_aastaraamat_veeb.

16. Saak’ashvili’s speech at the National Security Council after prison riots in Tbilisi, March 2006, https://old.civil.ge/eng/article.php?id=12191.

17. Saak’ashvili’s speech, 2006.

18. Georgian government response to CPT report, 2010, 9, https://rm.coe.int/CoERMPublicCommonSearchServices/DisplayDCTMContent?documentId=09000016806961de.

19. Georgian response to CPT report, 2010.

20. Georgian government response to CPT report, 2014, 3, https://rm.coe.int/CoERMPublicCommonSearchServices/DisplayDCTMContent?documentId=09000016806961f9.

21. At the time of writing – summer 2023 – the government is still led by Georgian Dream.

22. Male, late 30s, interviewed in Estonia, July 2020.

23. The CPT and Georgia, Latest news (2021), https://www.coe.int/en/web/cpt/-/council-of-europe-anti-torture-committee-cpt-visits-georgia-and-holds-high-level-talks-with-the-minister-of-justice-on-the-situation-in-semi-open-penitentiary-establishments.

24. Male, 42, interviewed in Georgia, December 2020.

25. https://www.bbc.com/russian/features-47588135.

26. We did not, however, interview anyone who was relegated to the very bottom of this system. At the bottom of the informal prison hierarchy are the called *opushchennye*. These are individuals live separately from other inmates since they are considered to be of a lower status. They are exploited by other prisoners for various often degrading tasks. Perhaps if they had been a part of our sample, narratives about increased safety of these prisoners from exploitation and violence (including sexual violence) would have been presented in a more prominent way. However, individuals who have experience of serving sentences as a part of this group are difficult to access, often traumatized, and do not readily share their stories with researchers.

27. Male, early 50s, interviewed in Estonia, July 2020.

28. Male, 64, interviewed in Georgia, November 2020.

29. The CPT and Georgia; Estonian response to CPT, 2002.
